# Supplementary figures and images for: Novel SPEA Superantigen Peptide Agonists and Peptide Agonist-TGFαL3 Conjugate. In Vitro Study of Their Growth-Inhibitory Effects for Targeted Cancer Immunotherapy
Source: Int J Mol Sci. 2023 Jun 22;24(13):10507. doi: 10.3390/ijms241310507 (PMC10341475; doi:10.3390/ijms241310507)

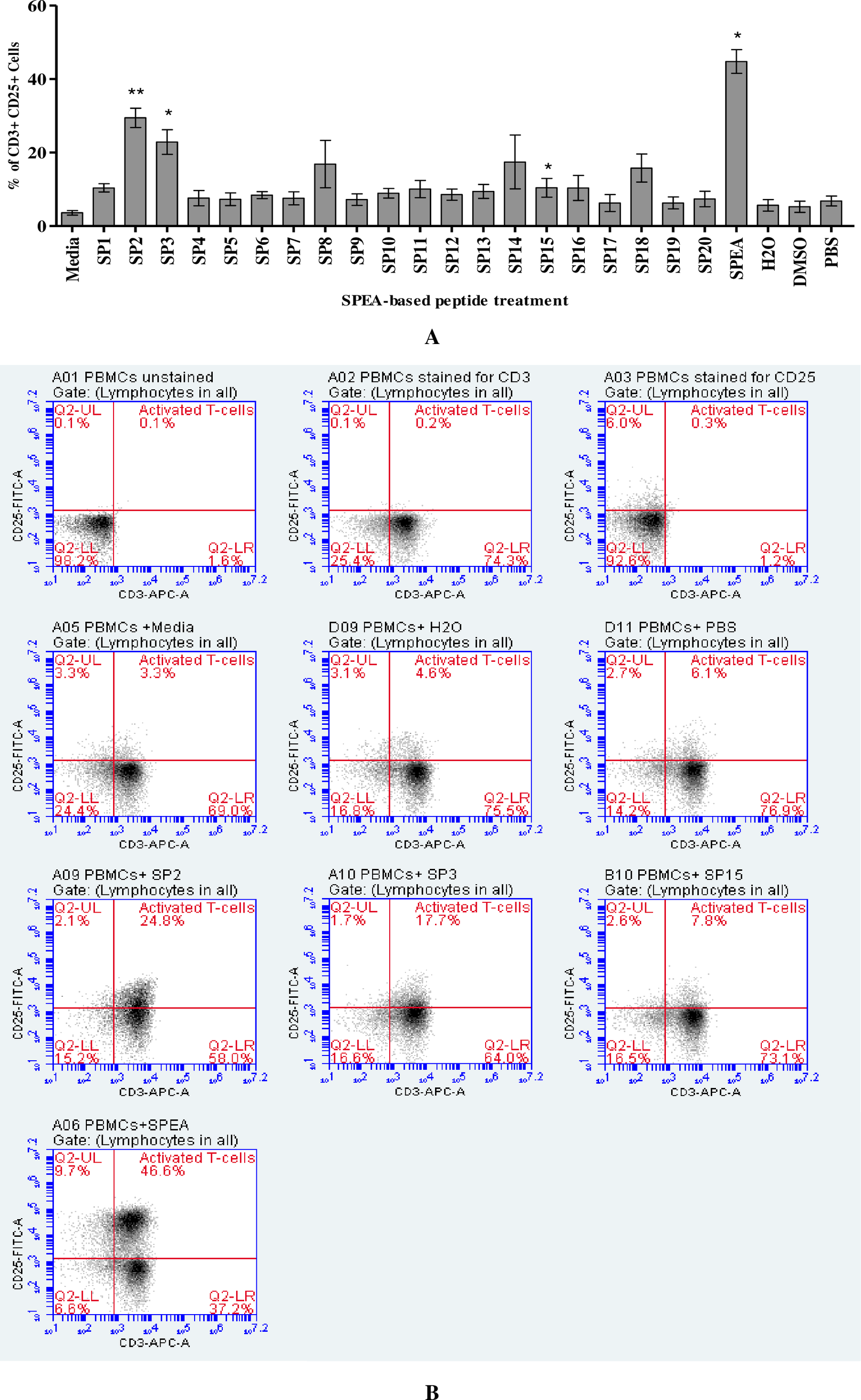

Supplement: Supplementary file 1 [file ijms-24-10507-s001.zip › PACE Corrected/fig1.tif]

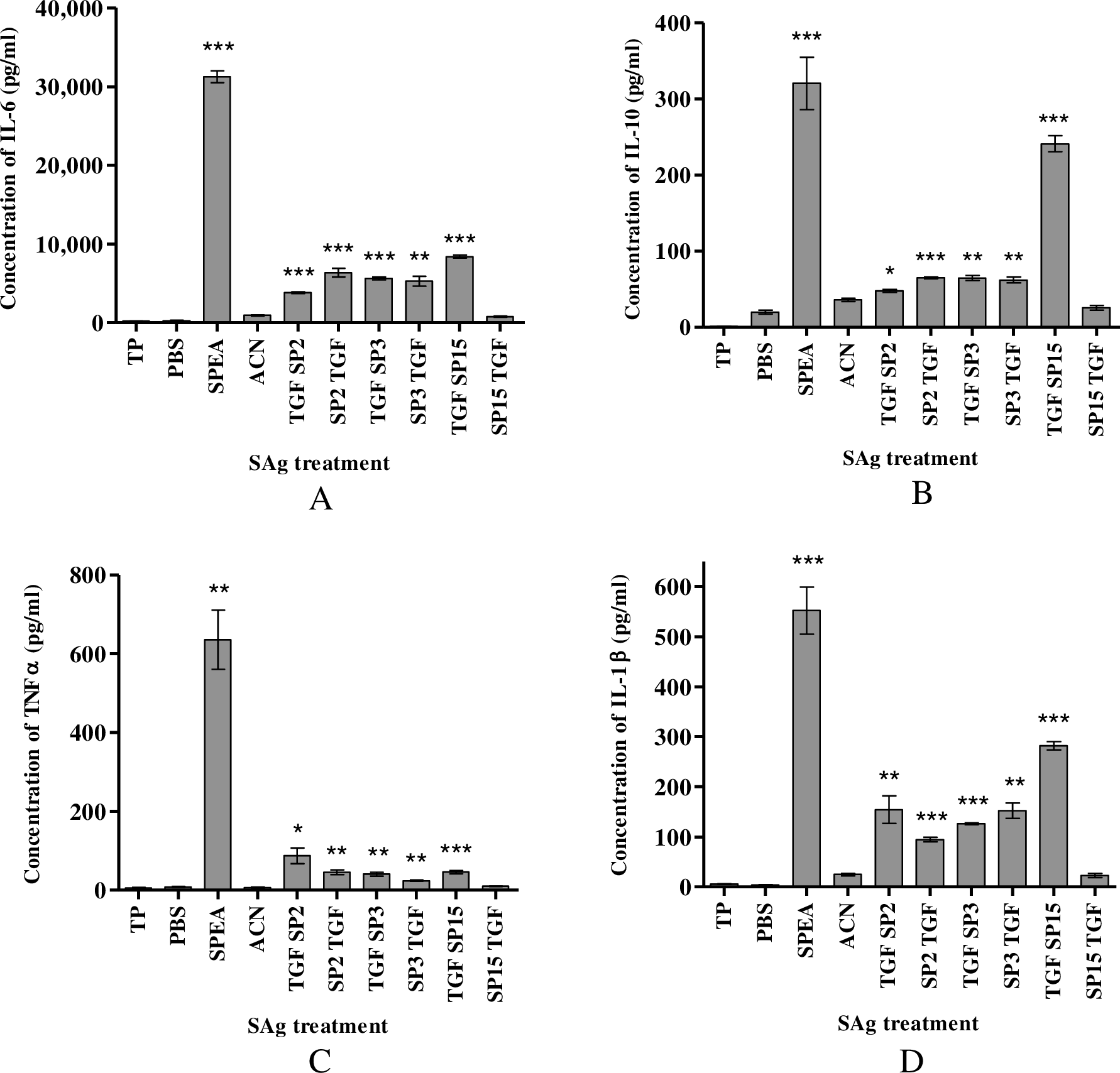

Supplement: Supplementary file 1 [file ijms-24-10507-s001.zip › PACE Corrected/fig3.tif]

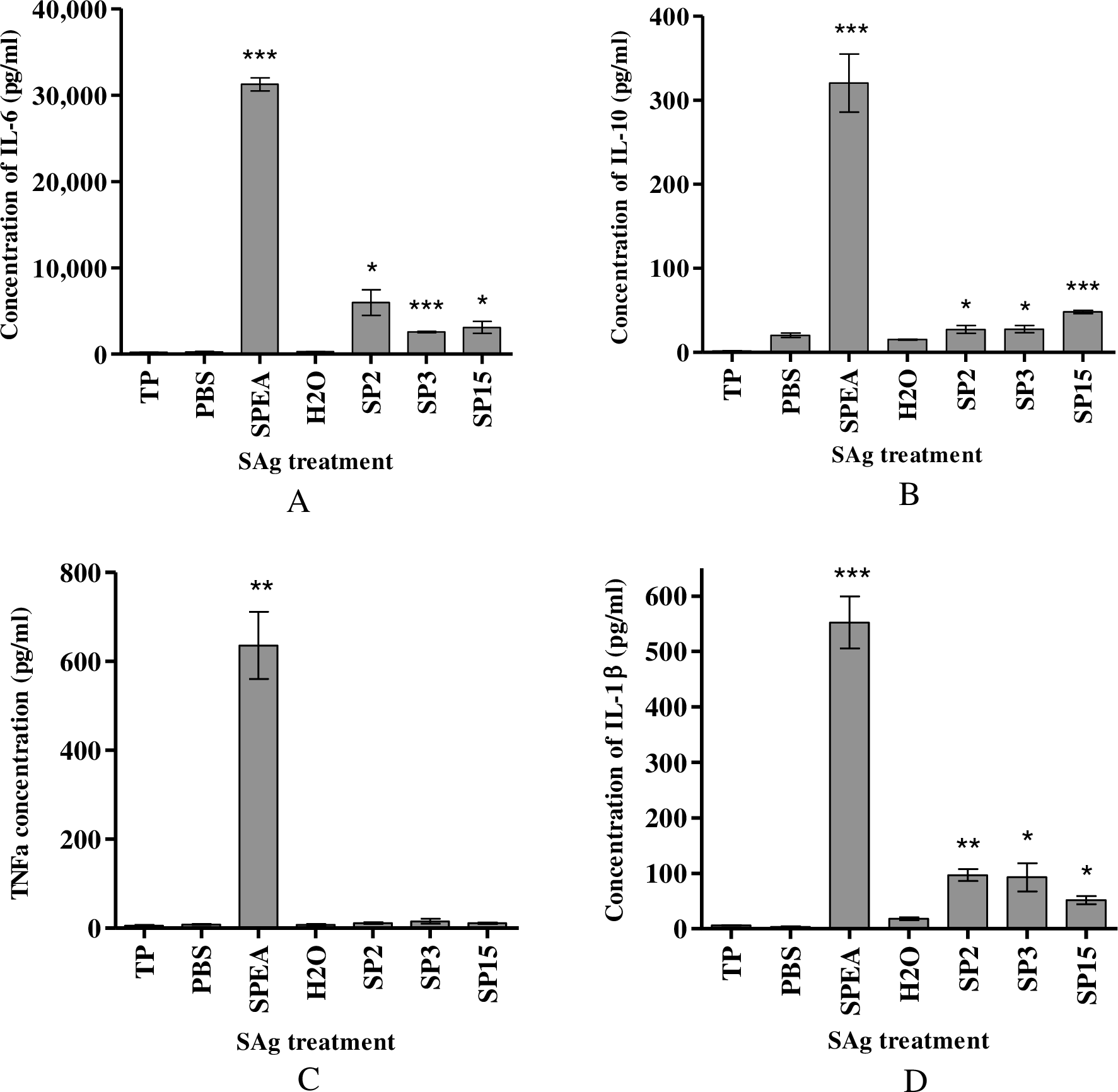

Supplement: Supplementary file 1 [file ijms-24-10507-s001.zip › PACE Corrected/fig2.tif]

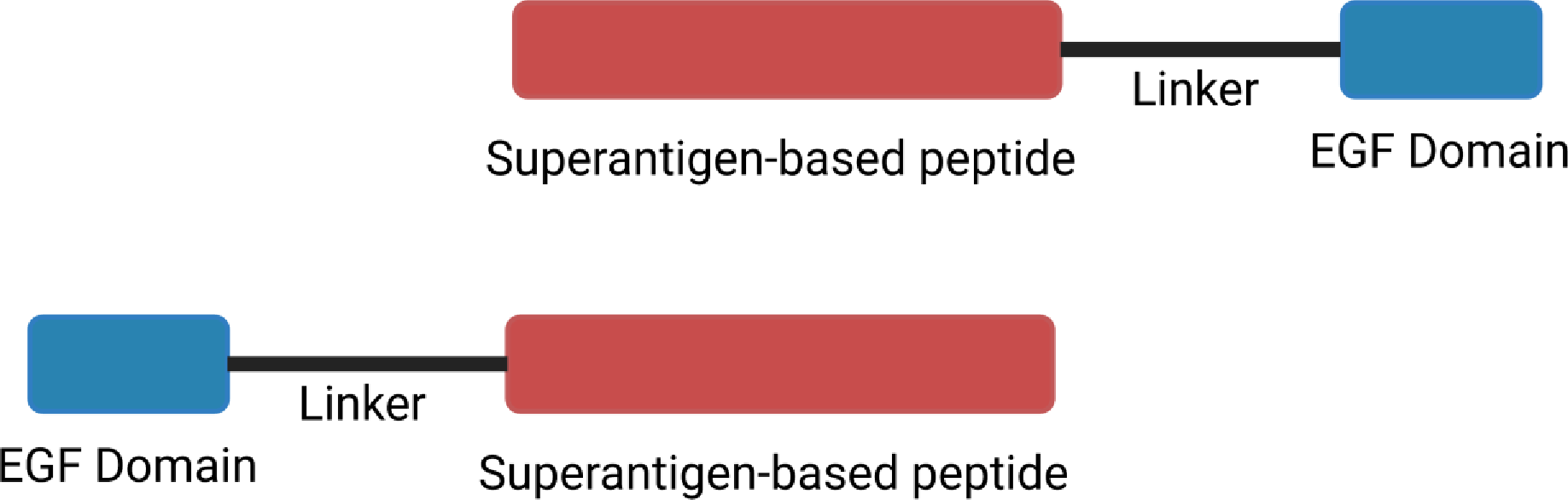

Supplement: Supplementary file 1 [file ijms-24-10507-s001.zip › PACE Corrected/fig8.tif]

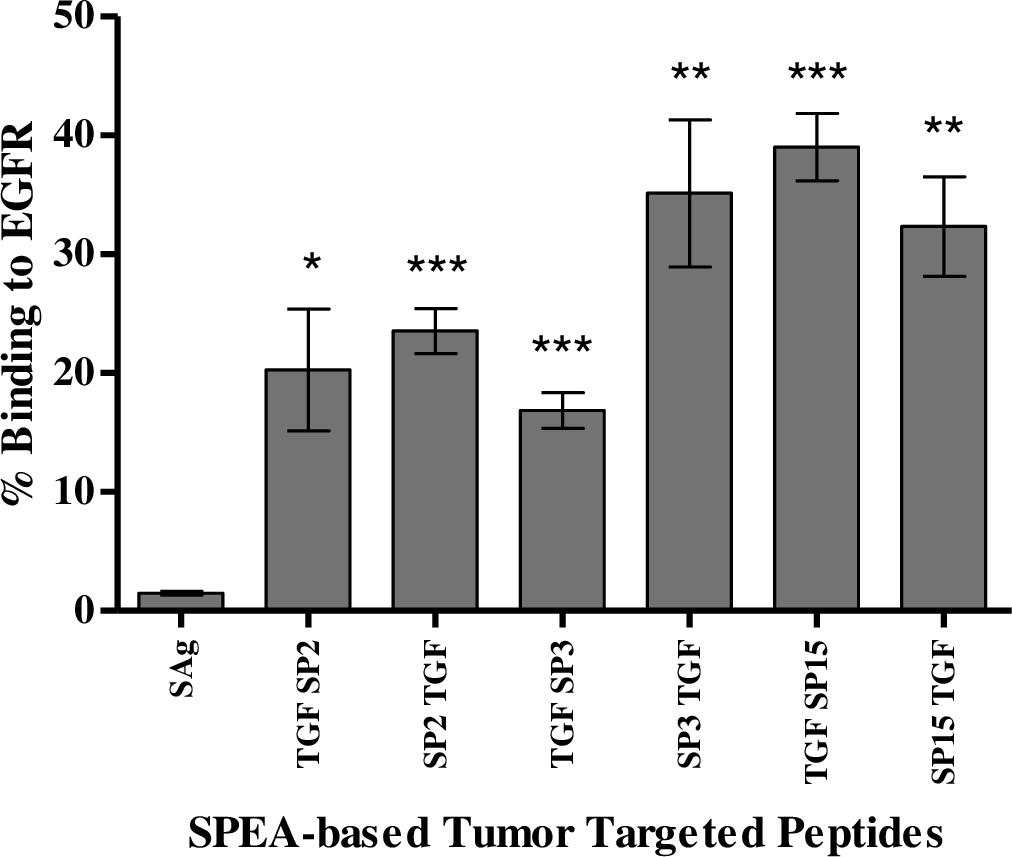

Supplement: Supplementary file 1 [file ijms-24-10507-s001.zip › PACE Corrected/fig4.tif]

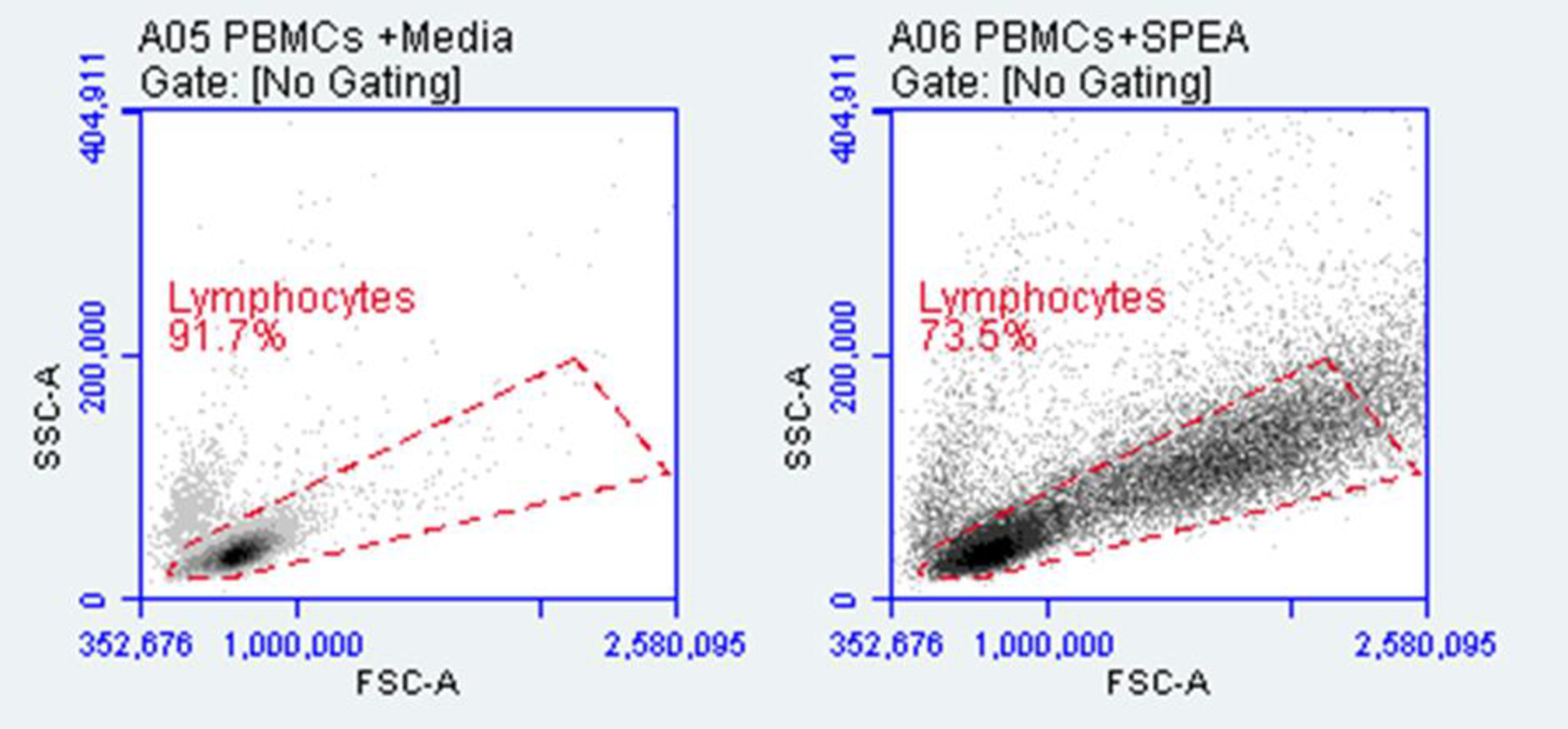

Supplement: Supplementary file 1 [file ijms-24-10507-s001.zip › PACE Corrected/Fig S1.tif]

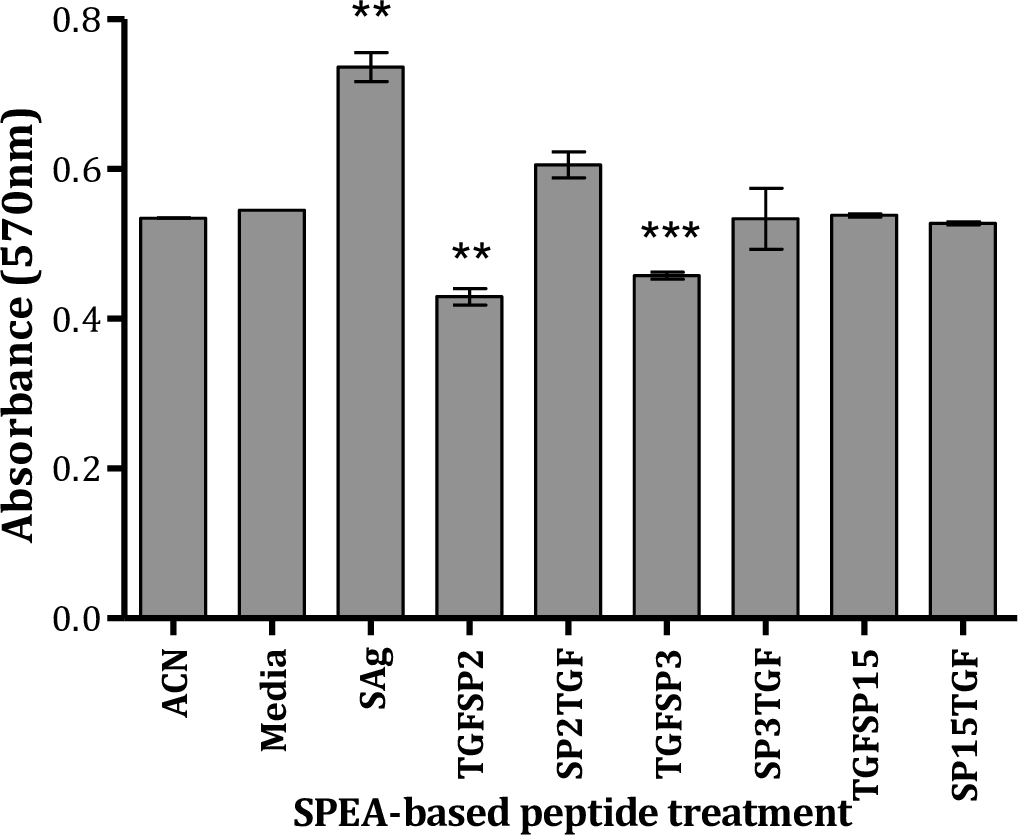

Supplement: Supplementary file 1 [file ijms-24-10507-s001.zip › PACE Corrected/fig5.tif]

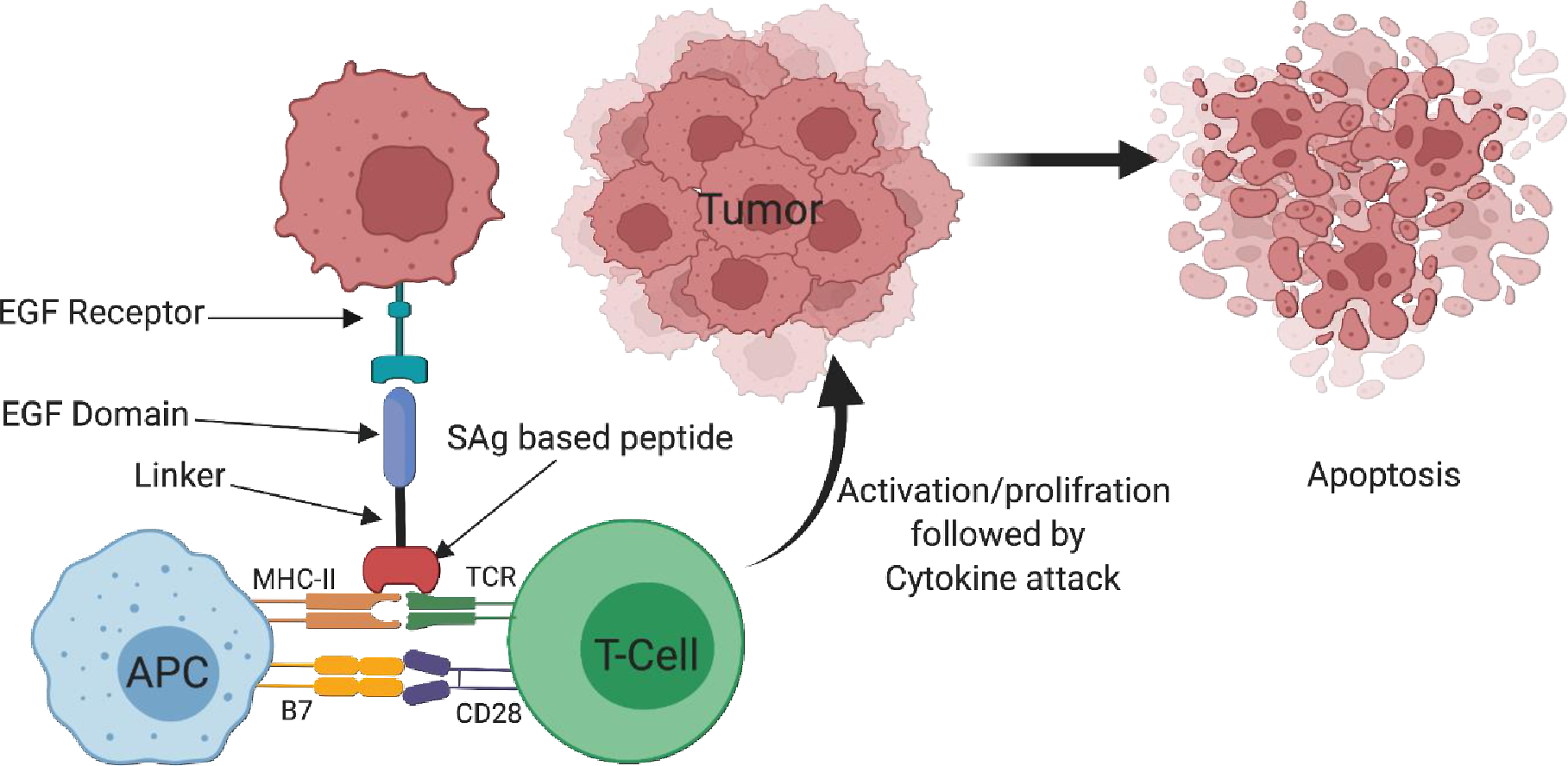

Supplement: Supplementary file 1 [file ijms-24-10507-s001.zip › PACE Corrected/fig9.tif]

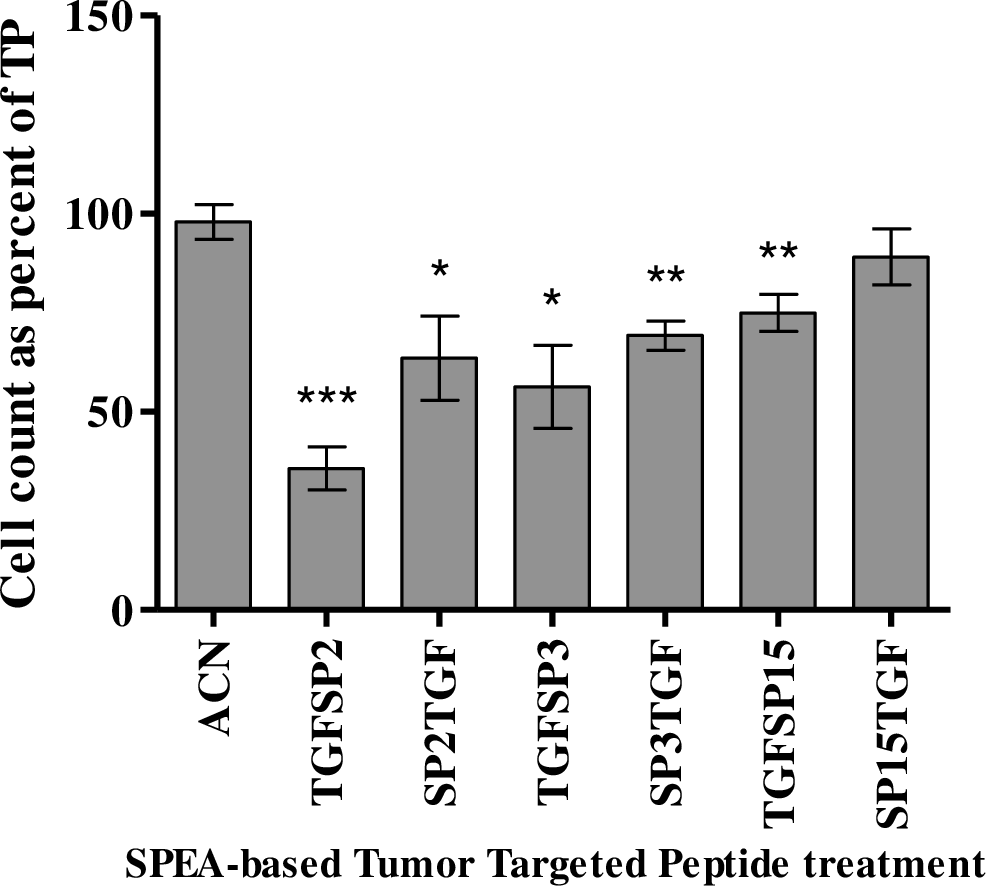

Supplement: Supplementary file 1 [file ijms-24-10507-s001.zip › PACE Corrected/fig7.tif]

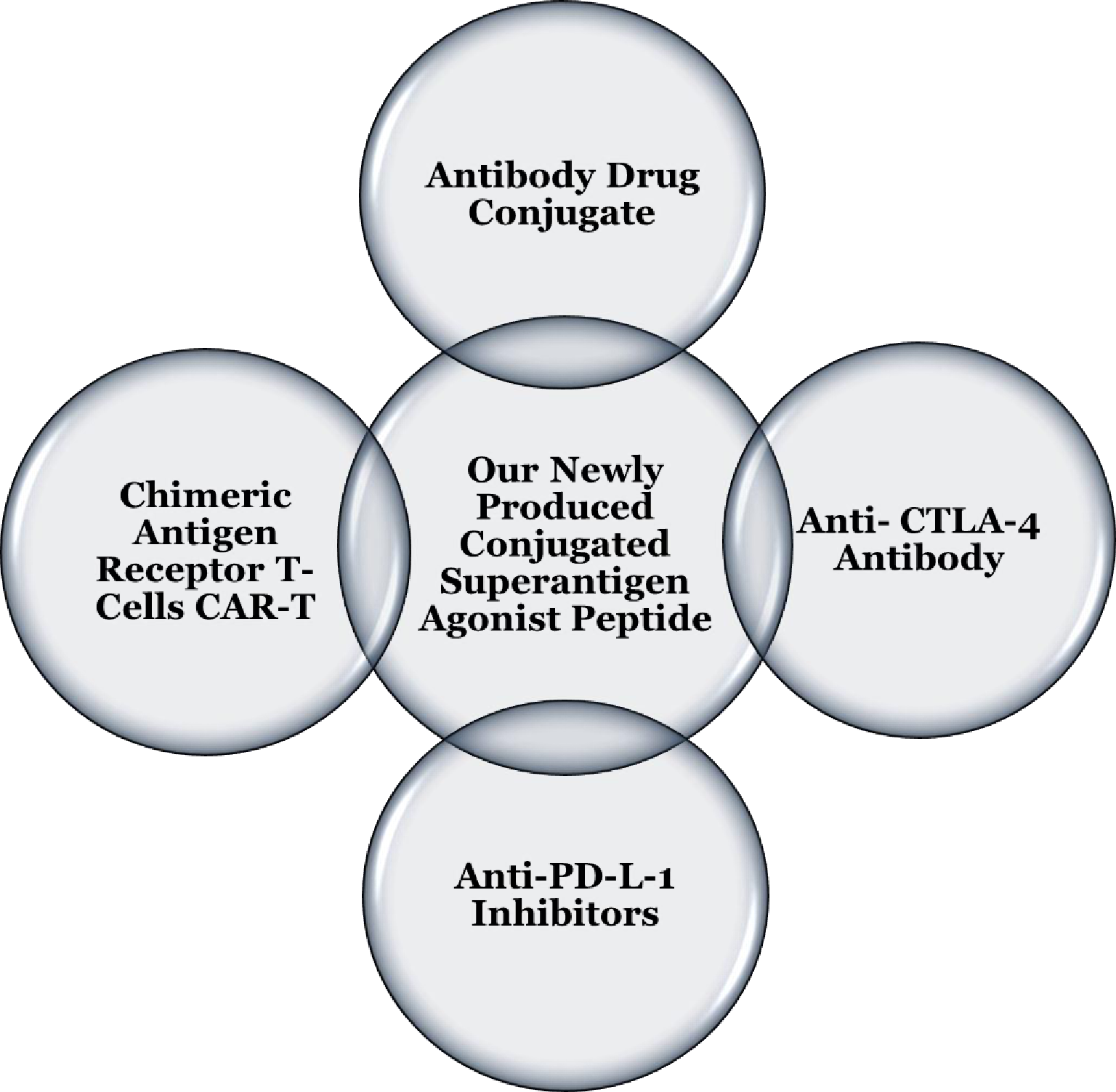

Supplement: Supplementary file 1 [file ijms-24-10507-s001.zip › PACE Corrected/fig10.tif]

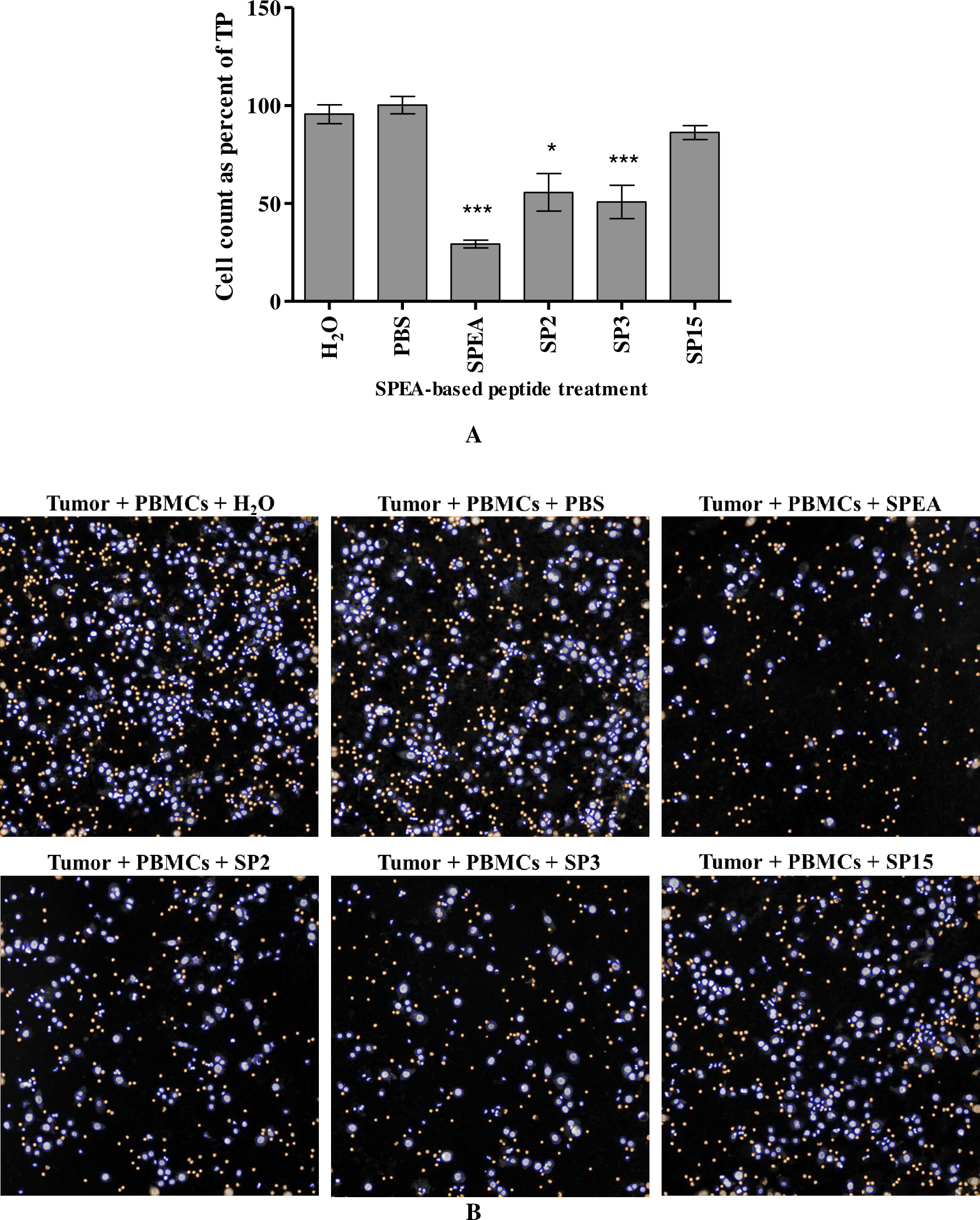

Supplement: Supplementary file 1 [file ijms-24-10507-s001.zip › PACE Corrected/fig6.tif]
